# Diversity and Metabolic Potential of the Terrestrial Mud Volcano Microbial Community with a High Abundance of Archaea Mediating the Anaerobic Oxidation of Methane

**DOI:** 10.3390/life11090953

**Published:** 2021-09-11

**Authors:** Alexander Y. Merkel, Nikolay A. Chernyh, Nikolai V. Pimenov, Elizaveta A. Bonch-Osmolovskaya, Alexander I. Slobodkin

**Affiliations:** Research Center of Biotechnology, Winogradsky Institute of Microbiology, Russian Academy of Sciences, Leninskiy Prospect, 33, Bld. 2, 119071 Moscow, Russia; alexandrmerkel@gmail.com (A.Y.M.); chernyh3@yandex.com (N.A.C.); npimenov@mail.ru (N.V.P.); elizaveta.bo@gmail.com (E.A.B.-O.)

**Keywords:** mud volcano, microbial communities, archaea, diversity, metagenome, methane, AOM, ANME, MAG

## Abstract

Terrestrial mud volcanoes (TMVs) are important natural sources of methane emission. The microorganisms inhabiting these environments remain largely unknown. We studied the phylogenetic composition and metabolic potential of the prokaryotic communities of TMVs located in the Taman Peninsula, Russia, using a metagenomic approach. One of the examined sites harbored a unique community with a high abundance of anaerobic methane-oxidizing archaea belonging to ANME-3 group (39% of all 16S rRNA gene reads). The high number of ANME-3 archaea was confirmed by qPCR, while the process of anaerobic methane oxidation was demonstrated by radioisotopic experiments. We recovered metagenome-assembled genomes (MAGs) of archaeal and bacterial community members and analyzed their metabolic capabilities. The ANME-3 MAG contained a complete set of genes for methanogenesis as well as of ribosomal RNA and did not encode proteins involved in dissimilatory nitrate or sulfate reduction. The presence of multiheme *c*-type cytochromes suggests that ANME-3 can couple methane oxidation with the reduction of metal oxides or with the interspecies electron transfer to a bacterial partner. The bacterial members of the community were mainly represented by autotrophic, nitrate-reducing, sulfur-oxidizing bacteria, as well as by fermentative microorganisms. This study extends the current knowledge of the phylogenetic and metabolic diversity of prokaryotes in TMVs and provides a first insight into the genomic features of ANME-3 archaea.

## 1. Introduction

Mud volcanism is one of the most exciting geological phenomena with significant implications for hydrocarbon exploration, seismicity, and the atmospheric budget of methane [1]. Terrestrial mud volcanoes (TMVs) arise due to high pressure in the deep sediment layers that results in the transport of mud, water and gas to the surface [2]. Mud and fluid emission structures are morphologically diverse, varying from small mud pools and salsa lakes to conical gryphons. The breccia, liquid and gas emissions from TMVs are geologically connected to deep subsurface petroleum and natural gas reservoirs, and to the deep biosphere. The presence of different inorganic and organic compounds, which can be used as electron donors and acceptors in microbial metabolism, allows for the development of microorganisms with various physiologies.

Microbial communities inhabiting submarine mud volcanoes have been extensively studied due to the proposed significant environmental impact of these ecosystems on the global methane cycle [3,4,5,6,7,8,9,10,11,12]. Microorganisms of terrestrial mud volcanoes have been investigated to a relatively small extent, a number of TMVs located in the areas of mud volcanism in Romania, Italy, Azerbaijan, Taiwan, China, Trinidad and Russia were inspected in relation to microbial community composition and abundance [13,14,15,16,17,18,19,20,21,22]. Metagenomic approaches were applied to explore the prokaryotic diversity of TMV only recently [23,24].

The prominent feature of all studied mud volcano microbial communities is the presence of anaerobic methane-oxidizing archaea (ANME). The numbers of ANME varies in different ecosystems, but usually ranges from 10^5^ to 10^7^ cell/g and accounts for 1–20% of the total microbial population [10,23]. These microorganisms, so far unavailable in pure culture, belong to the phylum *Euryarchaeota* (or to the phylum “Halobacteriota” according to GTDB [25] and are represented by three phylogenetic groups: ANME-1 with subgroups a and b (*Candidatus* Methanophagales), ANME-2 with subgroups a, b, c, d, and ANME-3. They are not monophyletic: ANME-2 and ANME-3 belong to *Methanosarcinales* while ANME-1 has a separate deep phylogenetic lineage [26,27]. Although ANME remains uncultivated, there are highly enriched cultures in *Candidatus* taxonomic status belonging to the “Methanoperedenaceae” family (formerly ANME-2d group or AOM-associated archaea (AAA) [28]. The biological mechanisms and biochemical pathways of anaerobic methane oxidation (AOM) by ANME are not fully understood. Different ANME groups can combine methane oxidation with the reduction of various electron acceptors, while the final stage of reduction can be carried out either by the ANME cells themselves or by a bacterial partner due to extracellular electron transfer. To date, the most investigated processes are the coupling of AOM with the reduction of sulfate, nitrate, nitrite, Fe (III) and Mn (IV) compounds [29,30,31,32,33].

The least studied ANME group is ANME-3. ANME-3 has been found mainly in marine ecosystems, including submarine mud volcanoes, deep-marine seeps, shallow and coastal sediments [3,6,7,26,34,35,36,37]. There is a single study report on the detection of ANME-3 in basaltic and granitic rock samples from the deep terrestrial subsurface [38]. The electron acceptors that ANME-3 utilize during AOM are unknown. There are some indications that ANME-3 archaea could oxidize methane without a bacterial partner, probably due to direct electron transfer to Fe(III) or sulfate [35,36]. Nevertheless, the association of ANME-3 archaea with sulfate-reducing bacteria has also been demonstrated [37]. The reconstruction of the ANME-3 metabolism is hampered by the lack of the genomic data, which are still represented by only a small genome fragment in the fosmid library [39].

The metabolism of bacteria involved in AOM is poorly characterized. None of the ANME bacterial partners is available in pure culture, possibly because some of them are obligate syntrophs. In sulfate-reducing AOM consortia, ANME-1 and ANME-2 archaea are associated with bacteria known as SEEP-SRB1 or SEEP-SRB2 (*Desulfobacterota*). Metagenomic methods have shown that SEEP-SRB1 genomes contain multiheme cytochromes, but do not have the genes encoding periplasmic hydrogenases and formate dehydrogenases typical for free-living sulfate-reducing bacteria [40,41]. On the other hand, the bacterium *Candidatus* Desulfofervidus auxilii, involved in thermophilic AOM, can grow without ANME-1 archaea, using hydrogen as an electron donor for dissimilatory sulfate reduction [42]. The physiology of the bacteria associated with ANME-3 has not been studied.

During ongoing investigations of the microbial diversity in TMVs, we found an unexpectedly high abundance of 16S rRNA gene sequences of ANME-3 archaea in one of the studied samples. Here, we present the results on the assembled metagenome of this microbial community. Analysis of the recovered high-quality metagenome-assembled genomes (MAGs) of archaeal and bacterial community members allowed us to reveal their core metabolic capabilities and to get insight into the metabolism of anaerobic methanotrophs of ANME-3 group.

## 2. Materials and Methods

### 2.1. Sampling Sites Description, Sample Collection and Chemical Analysis

Mixed sample of sediment and water was collected in May 2017, from a salsa lake of terrestrial mud volcano Karabetova Gora (45.202 N, 36.783 E), Taman Peninsula, Russia. Sample was taken at a depth of 10–20 cm below the surface of the water into sterile 50 mL tube and transported to the laboratory, where it was prepared for microbiological and geochemical studies.

Temperature and pH of the sample were measured at the point of collection using a digital pH meter equipped with thermocouple (AZ Instrument). Concentrations of chloride and sulfate were analyzed in water extracts by HPLC with a Stayer ion chromatograph (Aquilon) with an IonPack AS4-ASC column (Dionex) and conductivity detector; the eluent was bicarbonate (1.36 mM)/carbonate (1.44 mM) and the flow rate was 1.5 mL min^−1^. Content of Fe(III) and Fe(II) in the mud (porewater and solid phase) was determined after 24 h extraction in 0.6 N HCl followed by colorimetric assays of Fe(III) with thiocyanate and Fe(II) with 2,2′-dipyridyl [43].

### 2.2. Radioisotopic Experiments

Methane oxidation rate was measured using ^14^C-methane (specific activity 1.16 GBq/mmol, JSC Isotope, Russia; 1 µCi per sample) dissolved in sterile degassed distilled water. Sediment samples (2.5 mL) in three replicates were collected into 5 mL cut-off syringes and sealed with a gas-tight butyl rubber stopper without headspace. ^14^C-methane (0.2 mL) was injected through the stopper and distributed evenly throughout the syringe. The samples were incubated for 120 h at 20 °C and fixed with 0.5 mL 2 M KOH. The sediment samples fixed prior to addition of the labeled methane were used as the controls. The samples were then treated as described previously [44].

### 2.3. DNA Extraction, 16S rRNA Gene Amplicon Sequencing and Analysis

DNA from mud samples was isolated using FastDNA Spin Kit for Soil according to the manufacturer’s protocol (MP Biomedicals, Santa Ana, CA, USA). The 16S rRNA amplicon libraries were prepared using PCR with universal primers to the V3–V4 region in accordance with the previously described technique [45]. The following primer system was used: 341F (5′-CCTAYGGGDBGCWSCAG-3′) [46] and Pro-mod-805R (5′-GACTACNVGGGTMTCTAATCC-3′) [47]. Sequencing was carried out on a MiSeq system (Illumina, USA) using the reagent kit, which can read 300 nucleotides from each end. Demultiplexing, as well as subsequent treatment and analysis of the sequences, was carried out using the relevant scripts of the QIIME 2 ver. 2019.1 software package [48]. The amplicon sequence variant (ASV) table was constructed using Dada2 script [49] and the SILVA 132 database [50]. All the sequencing data are deposited in NCBI BioProject PRJNA722162.

### 2.4. Quantitative Polymerase Chain Reaction (qPCR)

For quantification of ANME-3 microorganisms in the samples, a primer system for qPCR was designed using Primer-BLAST [51] and obtained sequences of V3–V4 region of 16S rRNA gene of ANME-3 microorganisms from Karabetova Gora TMV. Sequences of the resulted primers were ANME-3-16S-F 5′-ACTTTTATTGGGTCTAAAGG-3′ and ANME-3-16S-R 5′-AGTCTAACAGTATCCCCTGA-3′. The specificity of the obtained primer system was verified by TestPrime 1.0 software [52] as well as by cloning and sequencing of the PCR product. For calibration curve linearized plasmid pAL2-T (Evrogen, Moscow, Russia) with cloned ANME-3 16S rRNA gene fragment was used. qPCR analyses were carried out on a StepOnePlus Real-Time PCR System (ThermoFisher Scientific, Waltham, MA, USA) and qPCRmix-HS SYBR (Evrogen, Russia). For total prokaryotes quantification the following primer system was used: 341F (5′-CCTAYGGGDBGCWSCAG-3′) [46] and Pro-mod-805R (5′-GACTACNVGGGTMTCTAATCC-3′) [47]. For calibration curve genomic DNA of *Melioribacter roseus* P3M-2 was used. Standards, samples and negative controls were run in triplicate.

### 2.5. Metagenome Library Preparation, Sequencing and Analysis

A shotgun metagenome library preparation and sequencing were done in Genoanalytica Ltd., Moscow, Russia using Nextera XT Library Preparation Kit (Illumina, San Diego, CA, USA) according to the manufacturer’s protocol and HiSeq 2500 system (Illumina, San Diego, CA, USA) with the reagent kit, which can read 150 nucleotides from each end. Raw reads were processed with Trimmomatic [53] for adapter removal and quality filtering. Reads were then assembled using MetaSPAdes [54]. Raw and assembled reads have been submitted to JGI Genomes OnLine Database (GOLD Project ID Gp0394211). Reads were mapped back to the assembly using Bowtie2 software [55] for coverage calculation. Binning of contigs into the MAGs was carried out by MaxBin 2 [56], MetaBAT 2 [57] and CONCOCT [58]. Further aggregation and dereplication of these three sets of bins was carried out by DAS Tool [59]. Bin completeness and contamination were evaluated using CheckM [60]. Taxonomies were assigned to each bin using GTDBtk [61]. In the case when a more accurate phylogenetic analysis was needed, de novo phylogenetic trees were built using the list of 122 archaeal or 120 bacterial marker genes taken from GTDB [61]. These marker genes were identified in studied bins using Prodigal v2.6.3 [62], aligned using MAFFT v7.427 [63], trimmed using trimAl 1.2rev59 [64] and then concatenated. Phylogenetic trees were built using PhyML 3.0 [65] and the Bayesian-like transformation of approximate likelihood-ratio test for branches [66]. Substitution model for phylogenetic reconstruction was automatically selected by SMS algorithm [67]. LG [68] was selected as the best substitution model in all cases. Co-assembled metagenomes were submitted for gene calling and annotations through the RAST server and the DOE Joint Genome IMG-MER (Institute Integrated Microbial Genomes metagenomics expert review) pipeline [69,70]. Genes encoding metabolic functions were queried in RASTtk and IMG-MER using Blast search. A complete list of metabolic genes of interest that were queried in this study can be found in Supplementary Table S2. Possible autotrophy has been investigated in all MAGs. We studied KEGG (Kyoto Encyclopedia for genes and genomes) biochemical maps for seven known carbon fixation pathways in each MAG [71,72,73]. A complete list of KEGG identifiers for each of seven pathways can be found in addition in Supplementary Table S3.

## 3. Results and Discussion

### 3.1. Study Site and Geochemical Characteristics

**Figure 1:**
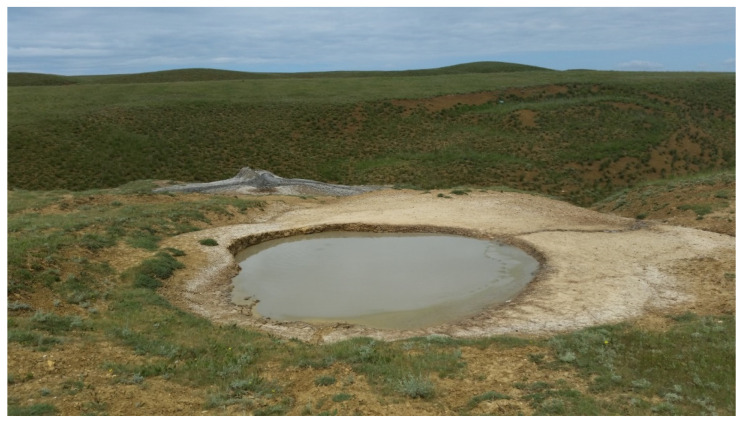
The image of the study site. A salsa lake at the top of Karabetova Gora mud volcano. The diameter of the lake is ca. 5 m.

The study site was a salsa lake (about 5.0 m in diameter) located in the top of the terrestrial mud volcano Karabetova Gora near the city of Taman (Figure 1). Karabetova Gora is 60 m high and is the biggest and one of the most active volcanoes of the Kerch-Taman mud volcanic province. The sampling site was actively venting; large and small gas bubbles were breaking the sediment surface with a relatively high frequency. Previous studies showed that gas from the TMVs of Taman province is mainly composed of methane (64–99%) accompanied by carbon dioxide and dinitrogen [74,75,76]. The sediment temperature was 20 °C. The water had pH 8.0 and contained 31.2 mM of chloride and 4.2 mM of sulfate. Dissolved sulfide, nitrate and nitrite were not detected (<0.01 mM). The water did not contain soluble iron ions, but the sediment had a high iron content (81.9 mM Fe(II) and 2.7 mM Fe(III)), due to the presence of iron-bearing minerals.

### 3.2. Microbial Communities Composition

First, the prokaryotic diversity was analyzed based on 16S rRNA gene amplicons high-throughput sequencing.

The total number of sequences obtained after filtering, denoising and chimera-checking by Dada2 was 130,574. The total number of unique sequences (ASVs) was 744. Chao1 values, calculated using an estimator of the true number of singletons [77], was 766. Thus, the coverage reached 97%. The Shannon index was 5.03, indicating the relatively high complexity of the studied microbial community

**Figure 2:**
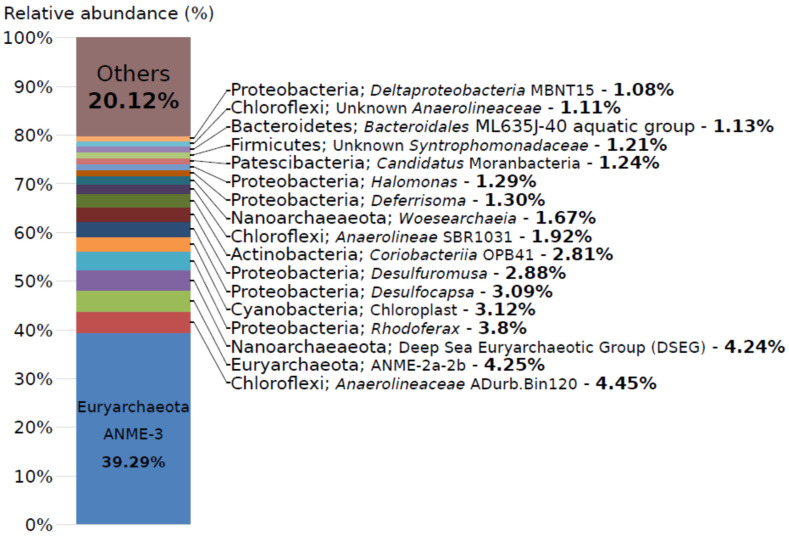
Relative abundance of taxonomic groups of prokaryotes in Karabetova Gora microbial community revealed by 16S rRNA gene amplicon sequencing. Only groups that are represented by more than 1% are shown. Classification is based on SILVA 132 rRNA databases. The lowest identified taxonomic levels are shown.

The prokaryotic population was almost equally represented by *Archaea* and *Bacteria* (51 and 49%, respectively). A characteristic feature of the studied microbial community was the explicit dominance of anaerobic methane-oxidizing archaea of ANME-3 group (39% of all reads) (Figure 2). Other archaeal sequences belonged to the anaerobic methane oxidizers ANME-2a-2b (4%) followed by the DSEG group (4%) and *Woesearchaeota* (2%). Methanogenic archaea were not abundant—sequences affiliated with the genus *Methanocalculus* composed 0.7%.

The dominant bacterial phylum was *Proteobacteria* (21% of all reads). Besides *Proteobacteria,* five other bacterial phyla constituted a considerable part in the studied community—*Chloroflexi* (9%)*, Bacteroidetes* (4%)*, Actinobacteria* (4%), *Firmicutes* (3%) and *Cyanobacteria* (3%). At the genus level, a substantial number of sequences belonged to uncultured *Anaerolineaceae* (8% of all reads) and to the members of the class *Deltaproteobacteria*—*Desulfocapsa* (3%), *Desulfuromusa* (3%) and *Deferrisoma* (1%) (Figure 2). Sequences of aerobic methylotrophic bacteria were detected only in a minor amount and were related to the genus *Methylomicrobium* (0.3%).

### 3.3. Abundances of 16S rRNA Genes

qPCR analysis was performed to assess the total number of prokaryotic cells, as well as to assess the number of cells of ANME-3 microorganisms in the studied community. The copy number of total prokaryotic 16S rRNA genes per 1ml of mud was estimated as 3.03 × 10^8^ (SD 9.01 × 10^6^) (R^2^–0.992; Eff%–54.3). Copy number of ANME-3 16S rRNA genes per 1ml of mud was estimated as 8.85 × 10^7^ (SD 1.15 × 10^7^) (R^2^–0.999; Eff%–89.5). Thus, the fraction of ANME-3 microorganisms in the microbial community estimated by the qPCR analysis was 29.21%.

### 3.4. Anaerobic Oxidation of Methane (AOM) Determined with Radiotracer Techniques

The potential in situ rate of AOM was measured directly in the original sample from a salsa lake over a period of 150 days after collection. The AOM activity was found to be 0.47 ± 0.03 nmol cm^−3^ day ^−1^ (± SD, *n* = 3). In the control experiment with the same sample that was fixed before the incubation methane removal, the rate did not exceed 0.02 ± 0.01 nmol cm^−3^ day ^−1^ (± SD, *n* = 3). The obtained rate of AOM in the Karabetova Gora mud volcano was in the range reported in several studies for marine and freshwater sediments [78].

### 3.5. MAGs General Characteristics and Phylogenetic Identification

A total of 33 MAGs with >50% completeness and <5% contamination were recovered from the metagenome. The MAGs statistics including number of contigs, genome size, presence of 16S rRNA gene and abundance among other MAGs calculated by reads mapping are shown in Table 1. The bins were assigned numerical identifiers in order of decreasing completeness. Of the 33 individual MAGs, 2 were 100% complete, 21 were 90–98% complete and 4 were 85–90% complete (estimated by CheckM). Additional characteristics of each bin, such as N50, GC content, number of predicted genes, presence of rRNAs and tRNAs, number of standard AA missing in tRNAs as well as GenBank Accession Number, are shown in the Supplementary Table S1.

According to phylogenomic analysis based on GTDB 06-RS202 [61], 29 of 33 MAGs were assigned to the domain *Bacteria*; and five were assigned to the domain *Archaea*. A complete (>1400 bp) 16S rRNA gene was found in 17 of 33 MAGs. Among *Bacteria,* the largest number of MAGs was assigned to the *Bacteroidota* phylum (7 MAGs), but with only 4.7% of the total abundance. The second largest number of MAGs was assigned to the *Desulfobacterota* phylum (five MAGs) with 9.4% of the total abundance. *Proteobacteria* were represented only by four MAGs with 2% of the total abundance. The most abundant bacterial phylum was “*Patescibacteria’*”(14%) represented mainly by “*Pacebacteria”* class members.

**Figure 3:**
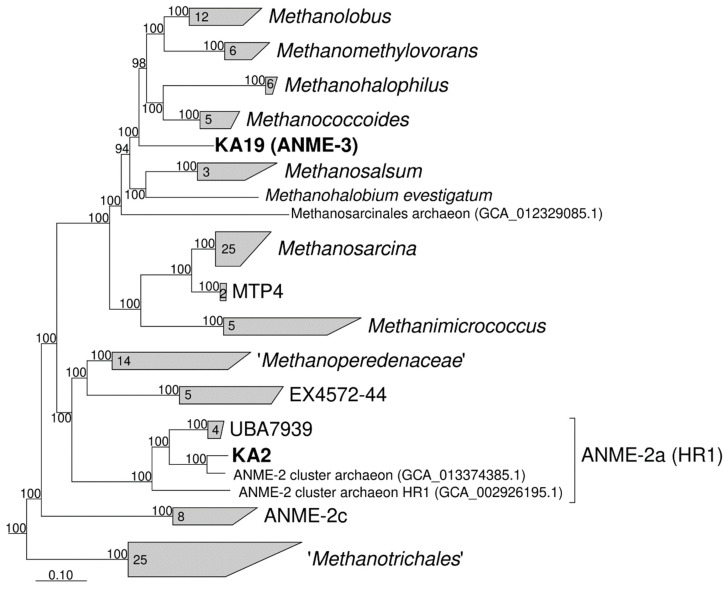
Phylogenomic placement of KA19 and KA2 MAGs based on concatenated partial amino acid sequences of 122 archaeal single copy conserved marker genes with taxonomic designations according to the GTDB. Bootstrap consensus tree is shown with values above 90% placed at the nodes. Bar, 0.1 changes per position.

The values of MAG abundance were different from the results obtained by 16S rRNA gene amplicons high-throughput sequencing. However, the number of ANME-3 archaea, which accounted for 30.32% of total abundance was in full agreement with the results of the qPCR analysis. The ANME-3 group was represented only by one MAG (KA19) with ~93% completeness. This MAG can be considered as a high-quality draft considering low contamination value (0.65%), the presence of 16S and 23S rRNAs and only two standard AA missing in tRNAs. The 16S rRNA gene phylogeny (Supplementary Figure S1a) demonstrates that this microorganism is closely related to ANME-3 archaea detected in Haakon Mosby mud volcano, deep-sea sediments of Monterey Canyon, and some other habitats [3,4,79]. The *mcrA* gene of KA19 (Supplementary Figure S1b) phylogenetically belongs to «group f» cluster previously found to be associated with ANME-3 microorganisms [4,5,6,7,8,9,10,11,12,13,14,15,16,17,18,19,20,21,22,23,24,25,26,27,28,29,30,31,32,33,34,35,36,37,38,39,40,41,42,43,44,45,46,47,48,49,50,51,52,53,54,55,56,57,58,59,60,61,62,63,64,65,66,67,68,69,70,71,72,73,74,75,76,77,78,79,80]. The phylogenomic position of KA19 MAG based on 122 archaeal conservative single copy marker genes (Figure 3) shows that it represents a genus level lineage within *Methanosarcinaceae* family and forms a monophyletic cluster with *Methanococcoides*, *Methanohalophilus*, *Methanomethylovorans* and *Methanolobus* genera. The phylogenomic position of KA2 MAG, the second anaerobic methane-oxidizing archaeon identified in our metagenomic data is within the well-established cluster ANME-2a (HR1).

Among the archaea found in the Karabetova Gora mud volcano there were also two MAGs of the EX4484-52 cluster which accounted for 11% of the total prokaryotic abundance. MAGs from this phylum-level linage were previously found in ANME-1-dominated hydrothermal sediments of Guaymas Basin [81]. According to our 16S rRNA based phylogenetic analysis, these MAGs correspond to DSEG group ASVs (Supplementary Figure S2). The related 16S rRNA sequences were also detected in methane-oxidizing archaea-dominated deep-sea hydrocarbon seep [82]. The role of these archaea in the community and their relationship with ANME microorganisms remains to be determined.

### 3.6. Insights into the Energy Metabolism of ANME Archaea

The MAGs of ANME-3 (KA19) and ANME-2a (KA2) contained all genes related to the seven central steps of methanogenic pathway. Genes encoding formyl-methanofuran dehydrogenase (*fmd*, subunits A, B, C, D, F, G), formyl-methanofuran tetrahydromethanopterin formyl transferase (*ftr*), methenyl-tetrahydromethanopterin cyclohydrolase (*mch*), methylene-tetrahydromethanopterin dehydrogenase (*mtd*), methylene-tetrahydromethanopterin reductase (*mer*), tetrahydromethanopterin methyltransferase (*mtr,* subunits A, B, C, D, E, F, G, H), and methyl-coenzyme M reductase (*mcr*, subunits A, B, C, G) were identified (Supplementary Table S4). In KA2, two *ftr* homologs were present.

Genes encoding canonical hydrogenases of methanogens, such as ferredoxin-dependent hydrogenase (Ech), methanophenazine-dependent hydrogenase (Vho), F_420_-nonreducing hydrogenase (Mvh) and F_420_-dependent hydrogenase (Frh) were absent in KA19 and KA2. The enzymes responsible for methanol and methylamines utilization—methanol-corrinoid methyltransferases, mono-, di- and three-methylamine methyltransferases and methanol dehydrogenase were also not encoded in either ANME genomes (Supplementary Table S2). Thus, most probably, ANME-3 archaea use the methanogenic pathway for methane oxidation according to “reverse methanogenesis” hypothesis, as it was suggested for other ANME groups [83,84,85].

Electrons generated from methane oxidation could be transferred to the lipid-soluble electron acceptors, methanophenazine or menaquinone, via the energy-conserving Fpo, Hdr and Rnf complexes, which oxidize F_420_H_2_, CoM-SH + CoB-SH, and reduced ferredoxin respectively. Genes of F_420_H_2_:phenazine oxidoreductase (Fpo subunits A, B, C, D, H, I, J, K, L, M, N, F), cytoplasmic (HdrABC) and membrane bound (HdrDE) coenzyme B- coenzyme M heterodisulfide reductase and Na^+^-translocating, ferredoxin:NAD oxidoreductase (Rnf subunits A, B, C, D, G, E) were present in both MAGs. Methanophenazine and menaquinone have considerably different redox potentials that have implications for subsequent electron transport pathways. Previous studies indicate that ANME-2a archaea do not contain genes for menaquinone biosynthesis and probably use methanophenazine as the electron carrier [84], while *Methanoperedens*-like archaea encode the menaquinone biosynthesis pathway and most likely use menaquinone [86]. In the genomes of KA2 and KA19, we did not find gene domains specific for menaquinone biosynthesis. Similar to some other ANME-2a archaea, KA2 encoded a protein with a domain specific for PhzF, which is an enzyme involved in phenazine biosynthesis in *Pseudomonas fluorescens* [84]. In KA19 as well as in methanophenazine-containing *Methanosarcina barkeri* MS [86] the respective gene is not present. Therefore, we assume that ANME-3 archaea use methanophenazine as the membrane integral electron carrier.

The nitrate reductase complex responsible for nitrate reduction coupled to AOM in *Ca.* M. nitroreducens [87], was not encoded in either the KA19 or in KA2 genomes. No homologs of *nar, nap, nrf, nir, nor or nos* genes were found. Genes involved in dissimilatory sulfate reduction (*dsrAB, dsrC, qmoA, aprAB, sat*) were also absent. Hence, most likely both archaea do not use nitrate or sulfate as electron acceptors.

The presence of multiheme c-type cytochromes (MHCs) was noted in ANME-1 and ANME-2 microorganisms [88]. Apparently in the members of *Ca.* Methanoperedenaceae, MHC are involved in the reduction of insoluble Fe(III) or Mn(IV) oxides [24,32,33]. The presence of MHC in ANME-3 has not been reported. We identified 13 MHCs in the genome of KA19 and 28 MHCs in the genome of KA2 (Table 2, Supplementary Tables S5 and S6). Ten MHCs in KA19 genome and 23 MHCs in KA2 genome were predicted to contain C-terminal transmembrane helices, indicating they were localized on the external side of the cell membrane. The number of CxxCH heme *c* binding motifs in a single MHC varied from 2 to 15 in KA19 and from 2 to 67 in KA2.

Thus, we can hypothesize that in the studied microbial community, ANME-3 and ANME-2a archaea use metal oxides or a syntrophic metal- or sulfate-reducing bacterial partner as an electron acceptor for AOM.

### 3.7. Metabolic Capabilities in MAGs: CO_2_ Fixation

**Figure 4:**
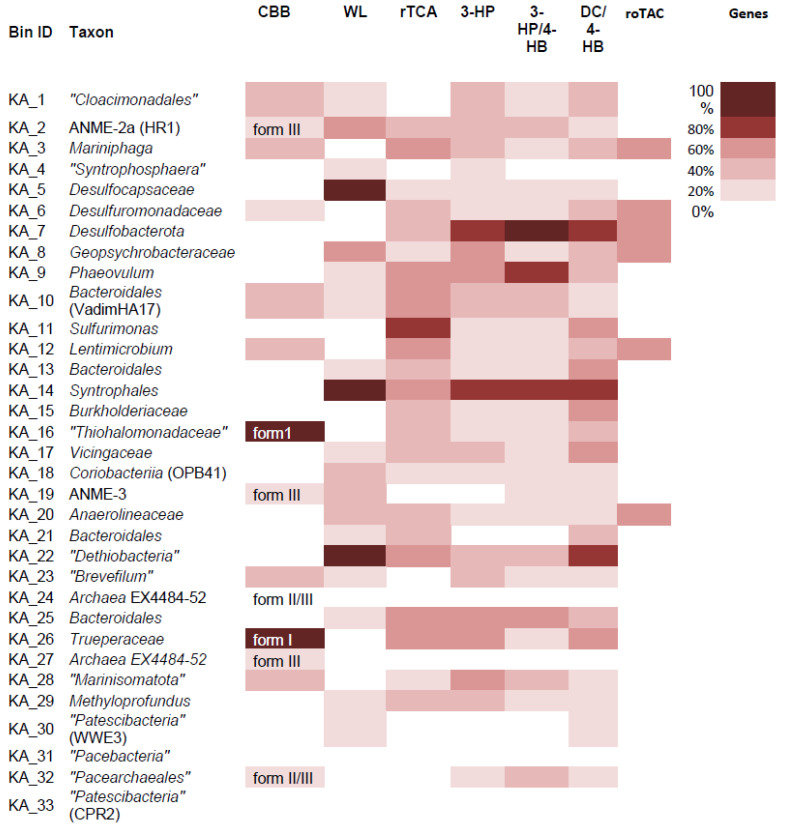
Identification of carbon fixation capabilities in all MAGs. Heat map indicates the percentage of essential genes present in seven well-characterized carbon fixation pathways. A full list of genes queried is described in Supplementary Table S3. CBB—the Calvin−Benson−Bassham cycle; WL—reductive acetyl-CoA synthase (Wood–Ljungdahl) pathway; rTCA—the reductive tricarboxylic acid cycle; 3-HP—3-hydroxypropionate bicycle; 3-HP/4-HB—3-hydroxypropionate/4-hydroxybutyrate cycle; DC/4-HB—dicarboxylate/4-hydroxybutyrate cycle; roTCA—reversed tricarboxylic acid cycle.

The possibility of carbon fixation was investigated in each of 33 MAGs (Figure 4). Six MAGs had full genomic potential for CO_2_ assimilation and could belong to autotrophic microorganisms. At the same time, some enzymes of the pathways of CO_2_ fixation could be used during heterotrophic growth. The reductive acetyl-CoA (Wood-Ljungdahl) pathway was the most frequent; three MAGs (*Desulfocapsaceae, Syntrophales, “Dethiobacteria”*) contained all the essential genes involved in this pathway (the major genes are listed in the Supplementary Table S3). The Wood−Lyngdahl pathway can also be used to assimilate various C_1_ compounds such as CO, formaldehyde, methanol, methylamine, methyl mercaptan, and methyl groups of aromatic O-methyl ethers/esters. The functioning of this pathway requires strict anoxic conditions, since some of its enzymes, especially CO dehydrogenase/acetyl-CoA synthase, are very sensitive to oxygen. Two MAGs contained a complete set of genes encoding the enzymes involved in carbon fixation via the Calvin−Benson−Bassham (CBB) reductive pentose phosphate cycle. These MAGs belonged to “*Thiohalomonadaceae*” and *Trueperaceae*. There are currently no reports on the CBB cycle functioning in the members of *Trueperaceae* which belongs to the *Deinococcus-Thermus* phylum. The sequences of the RuBisCO genes in this MAG were homologous with the known type I RuBisCO that catalyzes carboxylation of ribulose 1,5-bisphosphate to two molecules of 3-phosphoglycerate [89]. Type III and type II/III RuBisCO were detected in five MAGs belonging to various lineages. These types of RuBisCO are usually involved in the metabolism of nucleotides and nucleosides, with the exception of type III in members of *Thermodesulfobium* and *Ammonifex* where it participates in CO_2_ assimilation in the CBB cycle [90]. However, the completeness of the CBB cycle in MAGs with types III RuBisCO was low. The enzymes of the CBB cycle are completely resistant to molecular oxygen. The employing of a CBB cycle could be beneficial for microorganisms growing in microaerobic or aerobic conditions in the upper sediment layers of TMVs. Four other carbon fixation pathways (3-hydroxypropionate bicycle, 3-hydroxypropionate /4-hydroxybutyrate, dicarboxylate/4-hydroxybutyrate, and the reductive citric acid cycle) were less frequent. Surprisingly, one bacterial MAG (KA7; unknown *Desulfobacterota*) had all the necessary genes for the 3-hydroxypropionate/4-hydroxybutyrate cycle. So far, this pathway has only been found in archaea of the phylum *Crenarchaeota* [71]. The presence of genes encoding the characteristic enzymes of the 3HP/4HB cycle in mesophilic aerobic archaea (“*Thaumarchaeota*”) suggests that these numerous marine archaea also use this cycle. It should be noted that all enzymes of 3-HP/4-HB pathway in KA7 MAG are only homologues of the known archaean proteins (27–57% of aa sequence identity and more than 80% coverage), so the autotrophy of KA7 cannot be convincingly concluded. One more autotrophic CO_2_ fixation pathway—reversed tricarboxylic acid cycle (“roTCA”), was described recently [72,73]. Six MAGs contained the key enzyme of this cycle, citrate synthase, but other enzymes of TCA cycle in these MAGs were not completely represented. Therefore, most likely, the “roTCA” cycle is not operative in any of the MAGs.

### 3.8. Metabolic Capabilities in MAGs: Electron Acceptors and Donors

In the studied microbial community, AOM could be coupled to Fe(III), nitrate or sulfate reduction. The biochemical mechanisms and pathways of dissimilatory Fe(III) reduction are diverse, and currently no universal genomic determinants of this process are known. In many organisms, c-type cytochromes play a key role in electron transfer out of the cell to the extracellular electron acceptor [91]. In contrast, the biochemical machinery of nitrate and sulfate reduction are well established. The presence of the selected functional genes related to the energy metabolism in the MAGs is shown in Table 3. The genes encoding for the catalytic subunits of dissimilatory nitrate reductase complexes of Nap or Nar type (*napA, narG*) were identified in 4 of 33 MAGs (*Desulfocapsaceae, Desulfobacterota* KA7, *Sulfurimonas*, *Vicingaceae*). The terminal enzyme of the complete denitrification, nitrous oxide reductase (NosZ), was encoded in three MAGs.

Sulfate-reducing microorganisms were not abundantly represented, only one MAG of *Desulfocapsaceae* contained the genes for dissimilatory sulfite reductase (*dsrAB*). In previous studies, it was assumed that the sulfate-reducing partner of ANME-3 could be microorganisms of the *Desulfobulbus* genus [3,4,92]. None of these microorganisms were found in the studied community in a noticeable amount. Moreover, the abundance of KA5, the only MAG with *dsrAB*, was also quite low—3%, according to 16S rRNA profiling and 1.4%, according to metagenomics. This does not allow us to consider this microorganism as a main bacterial partner of ANME-3 in the studied community. This data is consistent with other studies, where ANME-3 cells were detected without any associations with bacterial partners and support the hypothesis that ANME-3 archaea could oxidize methane without a bacterial partner [5,35,36]. We cannot exclude that KA5 could be a syntrophic partner of ANME-2a archaea KA2.

The oxidative branch of the sulfur cycle was more prominent. Genes required for the oxidation of reduced sulfur compounds, such as sulfide, elemental sulfur, thiosulfate or sulfite (*sqr, sox, soe*) were present in 6 of 33 MAGs (*Phaeovulum, Sulfurimonas, “Thiohalomonadaceae”, Anaerolineaceae, Trueperaceae* and *Methyloprofundus)*. These sulfur compounds can be used as potential electron donors with nitrate or oxygen (or probably with other inorganic compounds such as Fe(III), Mn(IV) or Ar(V)) as electron acceptors, therefore supporting microbial growth. MAGs of *Phaeovulum, Sulfurimonas, “Thiohalomonadaceae”* and *Trueperaceae* encode complete or almost complete pathways for CO_2_ fixation and are most likely autotrophic microorganisms. The presence of *Sulfurimonas* has been noted in TMVs [24]. *Sulfurimonas crateris* was isolated from Taman TMV in anoxic conditions; it can grow by oxidation of HS^−^, S^0^ and S_2_O_3_^2−^ coupled to denitrification [93].

The majority of the community members were anaerobes, but 35% of the MAGs contained genes of the cytochrome *c* oxidase of *aa_3_*- or *cbb_3_*-types and could belong to aerobic or microaerophilic microorganisms. Dissolved atmospheric oxygen could penetrate into the upper layers of sediment, therefore the presence of O_2_-reducing enzymatic systems for detoxification or respiration could be advantageous. Aerobic methane oxidation was reflected by the presence of a particulate methane monooxygenase gene (*pmoA*) in one MAG belonging to *Methyloprofundus*.

Periplasmic Fe-Fe hydrogenases were present in 24% of MAGs, suggesting the importance of metabolic pathways connected to fermentation. Homologs of GH1 beta-glucosidase indicative of carbohydrate utilization were found in 15% of the MAGs, including uncultivated candidate taxa *“Brevefilum”* and *“Patescibacteria”*.

Other metabolic capabilities identified in the MAGs included the putative utilization of aromatic compounds and carbon monoxide (CO). The complete four subunits of the anaerobic aromatic-ring-reducing enzyme—Bzd-type benzoyl-CoA reductase, was encoded in two MAGs (*Desulfobacterota* KA7 and *Vicingaceae*). This enzyme is characteristic for facultatively anaerobic bacteria capable of aromatic compound utilization. Indeed, both Bzd-containing MAGs also contained genes of the NarG-type dissimilatory nitrate reductase. A gene cluster indicative of carbon monoxide oxidation under aerobic conditions (*coxMLS*) was found in the MAG of *Anaerolineaceae.* Genes encoding the catalytic subunit of anaerobic CO-dehydrogenase (*cooS*) were present in six MAGs, but only in one MAG (*Dethiobacteria*) *cooS* was localized in the cluster with *cooF*, the gene which encodes iron–sulfur-binding electron-transferring protein.

Therefore, the distribution of the functional genes in MAGs indicates that the bacterial members of the microbial community are mainly represented by autotrophic, nitrate-reducing, sulfur-oxidizing and fermentative microorganisms.

### 3.9. Genomic Features of Archaea EX4484-52:

KA24 and KA27 MAGs have very small genomes (1.16 Mbp and 1.08 Mbp, respectively) and an average amino acid identity of 49.58% between them. Phylogenetically, they are assigned to the Archaea EX4484-52 phylum-level lineage. According to GTDB 06-RS202 [61], this lineage is a part of DPANN superphylum and mostly related to *Nanohaloarchaeota*. One of distinctive genomic features of *Nanohaloarchaeota* is the presence of genes encoding very long (up to 8.553 amino acids) proteins called SPEARE [94]. These proteins promote attachment to the host cell and contain the serine protease, adhesion and restriction endonuclease domains. MAG KA27 encoded such a protein with a length of 12,127 amino acids (3.37% of the entire genome length). At both ends of this protein, there were transmembrane helices. It is one of the longest proteins currently known. MAG KA24 contained two such proteins, 8146 and 3105 aa in length. The former protein had a transmembrane helix at the N-terminus, and the latter protein had a transmembrane helix at the C-terminus.

## 4. Conclusions

The use of metagenomics allowed us to characterize a unique microbial community dominated by anaerobic methane-oxidizing archaea ANME-3 (39%). The high number of ANME-3 was further confirmed by qPCR. The measured AOM rate showed the activity of ANME in the given ecosystem. Thirty-three high-quality MAGs were recovered from the metagenome, among them the MAGs of archaea belonging to ANME-3, ANME-2a and EX4484-52 (DPANN) groups. The ANME MAGs contained a complete set of genes for the canonical methanogenic pathway, which most probably is used for methane oxidation according to “reverse methanogenesis” hypothesis. Proteins involved in dissimilatory nitrate or sulfate reduction were not encoded in ANME MAGs. We identified 13 MHCs in ANME-3 MAG and 28 MHCs in ANME-2a MAG indicating that these microorganisms can couple methane oxidation with the reduction of metal oxides or with the interspecies electron transfer to a bacterial partner. The presence of SPEARE proteins in EX4484-52 MAGs suggests that these archaea may be symbionts. Considering the high number of ANME-3 cells, they could be hosts of the EX4484-52 archaea. The bacterial population of the community was mainly represented by *Proteobacteria, Chloroflexi, Bacteroidetes, Actinobacteria, Firmicutes* and *Cyanobacteria*. The metabolic functions that could be assigned to these microorganisms include autotrophy, nitrate reduction, sulfur oxidation and the fermentation of organic compounds. Sulfate-reducing bacteria were not abundant, further confirming the assumption that in this ecosystem AOM does not depend on sulfate reduction. The most frequent autotrophic CO_2_ assimilation pathway was the Wood−Ljungdahl pathway.

Overall, our study extends the current knowledge of the phylogenetic and metabolic diversity of the prokaryotes inhabiting terrestrial mud volcanoes and presents a first analysis of the almost complete genome of ANME-3 archaea.

## Figures and Tables

**Table 1:** Overview of all genome bins >50% complete and with <5% contamination.

Bin ID	Domain	Taxon	Completeness %	Contami-nation %	# Contigs	Genome Size, Mbp	16S rRNA Gene, bp	Abundance, %
KA1	B	*“Cloacimonadales”*	100	0.00	52	2.39	1564	0.52
KA2	A	ANME-2a (HR1)	100	1.96	70	2.40	1470	1.72
KA3	B	*Mariniphaga*	98.92	3.23	305	5.12	1528	0.91
KA4	B	*“Syntrophosphaera”*	98.90	0.00	134	2.38	1553	0.45
KA5	B	*Desulfocapsaceae*	98.71	0.60	72	2.84	-	1.41
KA6	B	*Desulfuromonadaceae*	98.71	1.94	64	2.90	620	1.19
KA7	B	*Desulfobacterota*	98.32	0.84	54	2.62	1576	3.73
KA8	B	*Geopsychrobacteraceae*	98.06	0.65	61	2.75	686	1.71
KA9	B	*Phaeovulum*	97.88	1.06	69	2.86	-	0.75
KA10	B	*Bacteroidales* (VadinHA17)	97.86	3.35	263	3.39	403	0.41
KA11	B	*Sulfurimonas*	97.35	1.90	73	2.06	303	3.74
KA12	B	*Lentimicrobium*	97.31	1.61	108	4.19	-	0.98
KA13	B	*Bacteroidales*	97.22	1.61	224	3.57	-	0.96
KA14	B	*Syntrophales*	96.77	0.65	95	3.10	1569	1.34
KA15	B	*Burkholderiaceae*	96.30	3.47	509	3.72	1534	0.41
KA16	B	*“Thiohalomonadaceae”*	96.07	1.35	162	2.41	1420	0.46
KA17	B	*Vicingaceae*	95.16	0.54	232	3.66	1524	0.57
KA18	B	*Coriobacteria* (OPB41)	93.47	3.61	143	2.07	1523	1.36
KA19	A	ANME-3	92.81	0.65	275	1.80	1480	30.32
KA20	B	*Anaerolineaceae*	92.55	4.73	368	4.20	283	0.40
KA21	B	*Bacteroidales*	91.64	1.63	239	2.89	260	0.38
KA22	B	*“Dethiobacteria”*	90.25	2.26	170	2.36	-	0.43
KA23	B	*“Brevefilum”*	90.00	4.73	149	3.05	1525	1.20
KA24	A	*Archaea* EX4484-52	88.79	1.87	33	1.16	1468	2.10
KA25	B	*Bacteroidales*	88.17	2.96	382	4.18	-	0.51
KA26	B	*Trueperaceae*	87.92	1.55	389	3.29	1521	0.70
KA27	A	*Archaea* EX4484-52	85.98	1.87	20	1.08	1471	8.88
KA28	B	*“Marinisomatota”*	76.03	1.65	356	1.32	452	0.28
KA29	B	*Methyloprofundus*	73.30	1.92	384	1.96	-	0.28
KA30	B	*“Patescibacteria”* (WWE3)	73.12	0.16	71	0.84	497	0.37
KA31	B	*“Pacebacteria”*	71.24	1.12	52	0.98	1471	13.32
KA32	A	*“Pacearchaeales”*	69.39	0.00	9	0.64	1449	0.79
KA33	B	*“Patescibacteria”* (CPR2)	51.88	0.00	146	0.50	-	0.27

Domain: A—Archaea, B—Bacteria.

**Table 2:** Multiheme c-type cytochromes (MHCs), and components of sulfate and nitrate reduction systems encoded in MAGs of ANME and sulfate-reducing bacterium.

	KA19 (ANME-3)	KA2 (ANME-2a)	KA5 (*Desulfocapsaceae*)
Number of MHCs	13	28	26
Number of MHCs with CxxCH > 10	4	14	10
Maximal number of CxxCH in single MCH	15	67	16
Sulfate reduction genes	no	no	*dsrABD, dsrC, qmoABC, aprAB, dsrMKJOP*
Nitrate reduction genes	no	no	*napA, hcp*

Designations: *apr*—adenylylsulfate reductase; *dsr*, sulfite reductase, dissimilatory type; *hcp*—hydroxylamine reductase; *nap*—periplasmic nitrate reductase; *qmo*—sulfite reduction-associated complex.

**Table 3:** Metabolic potential of MAGs.

Bin ID	Taxon	Autotrophic Pathway	Genes: Electron Donors	Genes: Electron Acceptors	Putative Metabolism
KA1	*“Cloacimonadales”*			*Fe-hyd*	Fermentation
KA2	ANME-2a		*mcrA, cooS*		Anaerobic methane oxidation
KA3	*Mariniphaga*			*coxA, Fe-hyd*	Facultatively anaerobic
KA4	*“Syntrophosphaera”*			*Fe-hyd*	Fermentation
KA5	*Desulfocapsaceae*	WL	*cooS, hybC*	*napA, dsrAB*	Autotrophic, nitrate reduction, sulfite reduction, H_2_ utilization
KA6	*Desulfuromonadaceae*		*hybC*	*ccoN*	H_2_ utilization
KA7	*Desulfobacterota*	3-HP/4-HB	*bzd, hybC*	*coxA, ccoN, narG*	Autotrophic, nitrate reduction, H_2_ and aromatic compound utilization
KA8	*Geopsychrobacteraceae*		*cooS, hybC*	*coxA, ccoN*	Aerobic, H_2_ utilization
KA9	*Phaeovulum*		*GH1, sqr, soeA*	*coxA, ccoN, nosZ*	Aerobic, carbohydrates, sulfide and sulfite oxidation
KA10	*Bacteroidales*		*hybC*	*Fe-hyd*	Fermentation
KA11	*Sulfurimonas*		*soxYZ, sqr*	*ccoN, napA*	Sulfur compound oxidation, nitrate reduction
KA12	*Lentimicrobium*			*coxA, ccoN, Fe-hyd*	Facultatively anaerobic
KA13	*Bacteroidales*			*coxA, Fe-hyd*	Facultatively anaerobic
KA14	*Syntrophales*	WL	*cooS*		Autotrophic
KA15	*Burkholderiaceae*		*hybC*	*coxA, ccoN*	Aerobic, H_2_ utilization
KA16	*“Thiohalomonadaceae”*	CB	*soxB, sqr, soeA*	*ccoN*	Autotrophic, sulfur compounds oxidation
KA17	*Vicingaceae*		*bzd*	*coxA, ccoN, narG, nosZ*	Aerobic, denitrification, aromatic compound utilization
KA18	*Coriobacteria*		*hybC*		H_2_ utilization
KA19	ANME-3		*mcrA, cooS*		Anaerobic methane oxidation
KA20	*Anaerolineaceae*		*GH1, coxMLS, sqr, hyaB*	*coxA*	Aerobic, organotrophic, sulfur compound oxidation, H_2_ utilization
KA21	*Bacteroidales*			*nosZ*	Unidentified
KA22	*Dethiobacteria*	WL	*cooSF*		Autotrophic, acetogenic
KA23	*“Brevefilum”*		*GH1*		Carbohydrates utilization
KA24	Archaea EX4484-52				Unidentified
KA25	*Bacteroidales*			*coxA, Fe-hyd*	Facultatively anaerobic
KA26	*Trueperaceae*	CB	*sqr, hybC*	*coxA*	Autotrophic, sulfur compounds oxidation
KA27	Archaea EX4484-52				Unidentified
KA28	*“Marinisomatota”*			*Fe-hyd*	Fermentation
KA29	*Methyloprofundus*		*pMMO, sqr*	*coxA*	Aerobic methane and sulfide oxidation
KA30	*“Patescibacteria”*		*GH1*		Carbohydrate utilization
KA31	*“Pacebacteria”*		*GH1*		Carbohydrate utilization
KA32	*“Pacearchaeales”*				Unidentified
KA33	*“Patescibacteria”*				Unidentified

Names in brackets are based on uncultured representative. Designations of genes: *bzd*—benzoyl-CoA reductase, four subunits; *ccoN*—cytochrome *c* oxidase, *cbb_3_*-type; *coxA*—cytochrome *c* oxidase, *aa_3_*-type; *coxL*—carbon monoxide dehydrogenase (aerobic); *cooS*—carbon monoxide dehydrogenase (anaerobic); *dsrAB,* sulfite reductase, dissimilatory type; *GH1*—6-phospho- beta-glucosidase, glycoside hydrolase family 1; *Fe-hyd*—periplasmic [FeFe] hydrogenase; *hyaB*—periplasmic [Ni-Fe] hydrogenase large subunit*; hybC*—uptake hydrogenase, large subunit*; mcrA*—methyl coenzyme M reductase; *pMMO*—particulate methane monooxygenase; *napA*—periplasmic nitrate reductase; *narG*—respiratory nitrate reductase; *nosZ*—nitrous oxide reductase; *soeA*—sulfite dehydrogenase; *sox*—sulfur oxidation proteins; *sqr*—sulfide:quinone oxidoreductase.

## Data Availability

Not applicable.

## Supplementary Materials

The following are available online at https://www.mdpi.com/article/10.3390/life11090953/s1, Figure S1: Phylogenetic placement of ANME, Figure S2: Phylogenetic placement of EX4484-52 archaea, Table S1: Main characteristics of the MAGs, Table S2: The list of functional genes that were queried in this study, Table S3: The list of enzymes used to determine carbon fixation capability in MAGs, Table S4: Genes of methanogenic pathway in KA2 and KA19, Table S5: RAST annotation of KA19 and KA2, Table S6: Multiheme c-type cytochromes encoded in MAGs.
