# Supplementary material for: Diversity and Metabolic Potential of the Terrestrial Mud Volcano Microbial Community with a High Abundance of Archaea Mediating the Anaerobic Oxidation of Methane
Source: Life (Basel). 2021 Sep 11;11(9):953. doi: 10.3390/life11090953 (PMC8470020; doi:10.3390/life11090953)
Supplement: Supplementary file 1 [file life-11-00953-s001.zip › Supplementary Figures S1 and S2 for proof.pdf]

life

Supplementary material of Diversity and Metabolic Potential of the Terrestrial Mud Volcano Microbial Community with a High Abundance of Archaea Mediating the Anaerobic Oxidation of Methane

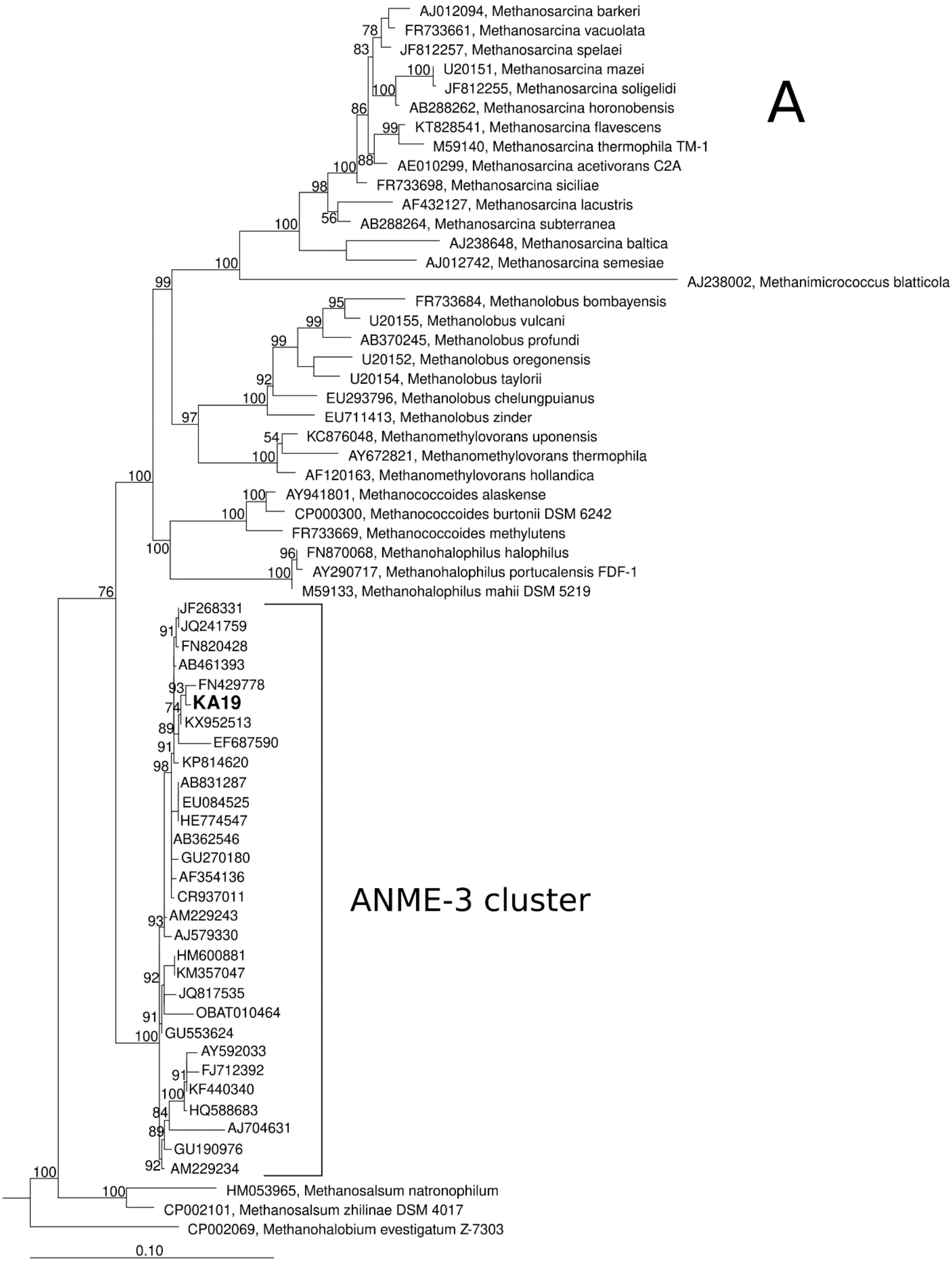
A
AJ012094, Methanosarcina barkeri
FR733661, Methanosarcina vacuolata
JF812257, Methanosarcina spelaei
U20151, Methanosarcina mazei
JF812255, Methanosarcina soligelidi
AB288262, Methanosarcina horonobensis
KT828541, Methanosarcina flavescens
M59140, Methanosarcina thermophila TM-1
AE010299, Methanosarcina acetivorans C2A
FR733698, Methanosarcina siciliae
AF432127, Methanosarcina lacustris
AB288264, Methanosarcina subterranea
AJ238648, Methanosarcina baltica
AJ012742, Methanosarcina semesiae
AJ238002, Methanimicrococcus blatticola
FR733684, Methanolobus bombayensis
U20155, Methanolobus vulcani
AB370245, Methanolobus profundi
U20152, Methanolobus oregonensis
U20154, Methanolobus taylorii
EU293796, Methanolobus chelungpuianus
EU711413, Methanolobus zinder
KC876048, Methanomethylovorans uponensis
AY672821, Methanomethylovorans thermophila
AF120163, Methanomethylovorans hollandica
AY941801, Methanococcoides alaskense
CP000300, Methanococcoides burtonii DSM 6242
FR733669, Methanococcoides methylutens
FN870068, Methanohalophilus halophilus
AY290717, Methanohalophilus portucalensis FDF-1
M59133, Methanohalophilus mahii DSM 5219
JF268331
JQ241759
FN820428
AB461393
FN429778
KA19
KX952513
EF687590
KP814620
AB831287
EU084525
HE774547
AB362546
GU270180
AF354136
CR937011
AM229243
AJ579330
HM600881
KM357047
JQ817535
OBAT010464
GU553624
AY592033
FJ712392
KF440340
HQ588683
AJ704631
GU190976
AM229234
ANME-3 cluster
HM053965, Methanosalsum natronophilum
CP002101, Methanosalsum zhilinae DSM 4017
CP002069, Methanohalobium evestigatum Z-7303
0.10

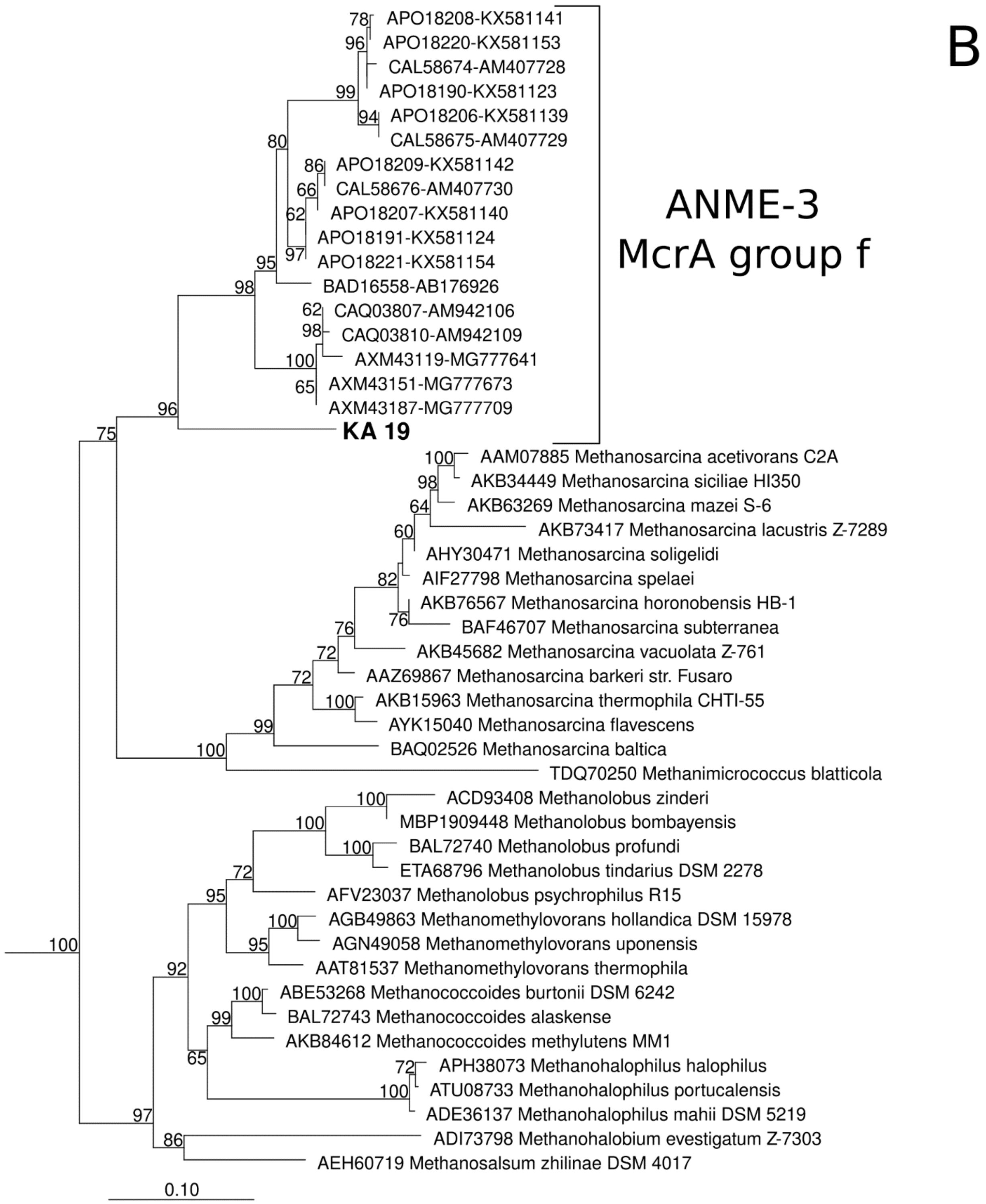

Figure S1. Phylogenetic placement of anaerobic methane-oxidizing archaea KA19 & KA2 based on the 16S rRNA gene sequences (A) or mcrA gene sequence (B). The tree was built using the maximum--likelihood method by the PhyML 3.0 program [Guindon et al., 2010] and the approximate likelihood-ratio test for branches [Anisimova et al., 2011]. Bootstrap values ≥50 % are shown at nodes. Bar, 0.10 changes per position. GenBank accession numbers are given in parentheses.

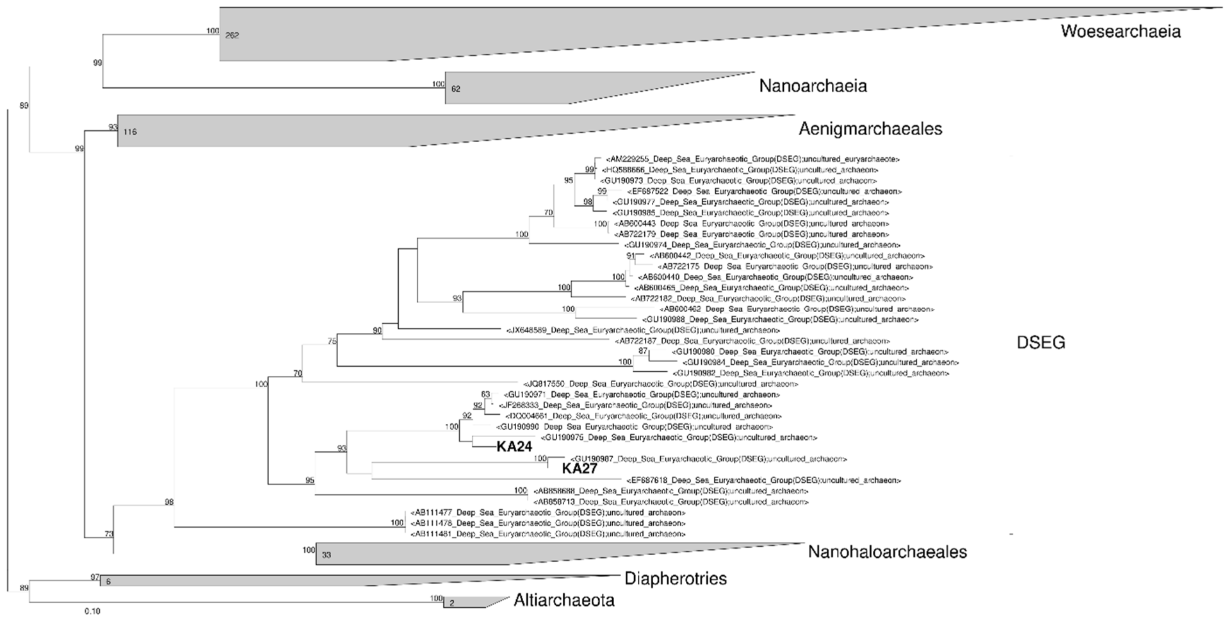

Figure S2. Phylogenetic placement of EX4484-52 archaea KA24 & KA27 based on the 16S rRNA gene sequences. The tree was built using the maximum--likelihood method by the PhyML 3.0 program [Guindon et al., 2010] and the approximate likelihood-ratio test for branches [Anisimova et al., 2011]. Bootstrap values ≥50 % are shown at nodes. Bar, 0.10 changes per position. GenBank accession numbers are given in parentheses.

Guindon S, Dufayard J-F, Lefort V, Anisimova M, Hordijk W et al. New algorithms and methods to estimate maximum-likelihood phylogenies: assessing the performance of PhyML 3.0. Syst Biol 2010;59:307–321.

Anisimova M, Gil M, Dufayard J-F, Dessimoz C, Gascuel O. Survey of branch support methods demonstrates accuracy, power, and robustness of fast likelihood--based approximation schemes. Syst Biol 2011;60:685–699.
