# Supplementary material for: Diversity and Metabolic Potential of the Terrestrial Mud Volcano Microbial Community with a High Abundance of Archaea Mediating the Anaerobic Oxidation of Methane
Source: Life (Basel). 2021 Sep 11;11(9):953. doi: 10.3390/life11090953 (PMC8470020; doi:10.3390/life11090953)
Supplement: Supplementary file 1 [file life-11-00953-s001.zip › Supplementary Table S3 Autotrophy for proof.pdf]

life

MDPI

Supplementary material of Diversity and Metabolic Potential of the Terrestrial Mud Volcano Microbial Community with a High Abundance of Archaea Mediating the Anaerobic Oxidation of Methane

Table S3. List of enzymes used to determine carbon fixation capability in MAGs.

Reductive Acetyl CoA	3-Hydroxypropionate bicycle	3-Hydroxypropionate/ 4-Hydroxybutyrate	Dicarboxylate/4-Hydroxybutyrate	Reductive Citric Acid	Calvin Cycle	Reversible tricarboxylic acid cycle
1.2.99.2*	1.3.4.1	6.4.1.3	2.7.9.1	CCS		
	1.3.1.6	1.2.1.76	2.7.9.2		4.1.1.39	
1.2.1.43	2.8.3.22	1.1.1.-	4.1.1.31	CCL		2.3.3.1
	5.4.99.2	6.2.1.40	1.1.1.37			
3.5.4.9	6.4.1.3	6.2.1.-	4.2.1.2	4.1.1.31	2.7.1.19	
	1.3.1.84	4.2.1.120	1.3.4.1			
1.5.1.20	42.1.116	4.2.1.17	1.3.1.6	1.3.1.6	2.7.1.14	
	6.2.1.36	1.1.1.35	2.8.3.22		4.1.2.13	
2.3.1.169	6.2.1.	6.4.1.2	1.2.1.76	2.7.9.1	3.1.3.37	
	1.1.1.298	1.2.1.75	1.1.1.-		4.1.2.13	
1.2.7.4	1.1.1.	1.1.1.298	6.2.1.40	2.7.9.2		
	1.2.1.75	6.2.1.36	6.2.1.-			
6.3.4.3	6.4.1.2	4.2.1.116	4.2.1.17	1.3.4.1		
	4.2.1.1.53	1.3.1.84	1.1.1.35			
1.5.1.5	5.4.1.3					
	4.1.3.24					
2.1.1.258	4.2.1.148					

* Kyoto Encyclopedia of Genes and Genomes (KEGG) identifiers
CCL = citryl-CoA lyaseCCS = citryl-CoA ligase

Reductive Acetyl CoA (Wood–Ljungdahl pathway)

1.2.99.2- carbon-monoxide dehydrogenase

1.2.1.43- formate dehydrogenase

3.5.4.9 - methenyltetrahydrofolate cyclohydrolase

1.5.1.20- methylenetetrahydrofolate reductase

2.3.1.169- CO-methylating acetyl-CoA synthase

1.2.7.4 - carbon-monoxide dehydrogenase

6.3.4.3 - formyltetrahydrofolate synthetase

1.5.1.5 - methylenetetrahydrofolate dehydrogenase

2.1.1.258- 5-methyltetrahydrofolate corrinoid/iron-sulfur protein Co-methyltransferase;

3-Hydroxypropionate bicycle

1.3.4.1 - fumarate reductase (CoM/CoB)

1.3.1.6 - fumarate reductase (NADH)

2.8.3.22 - uccinyl-CoA---L-malate CoA-transferase

5.4.99.2 - methylmalonyl-CoA mutase

6.4.1.3 - propionyl-CoA carboxylase

1.3.1.84- acrylyl-CoA reductase (NADPH)

42.1.116- 3-hydroxypropionyl-CoA dehydratase

6.2.1.36- 3-hydroxypropionyl-CoA synthase

6.2.1. - acetate---CoA ligase

1.1.1.298- 3-hydroxypropionate dehydrogenase (NADP+) RoseRS_3201

1.1.1. - With NAD+ or NADP+ as acceptor

1.2.1.75 - malonyl-CoA reductase (malonate semialdehyde-forming)

6.4.1.2 - acetyl-CoA carboxylase

4.2.1.1.53 - carbonic anhydrase

5.4.1.3 - 2-methylfumaryl-CoA isomerase

4.1.3.24 - malyl-CoA lyase

4.2.1.148 - 2-methylfumaryl-CoA hydratase

3-Hydroxypropionate/4-Hydroxybutyrate cycle

6.4.1.3 - propionyl-CoA carboxylase

1.2.1.76 - succinate-semialdehyde dehydrogenase

1.1.1. -With NAD+ or NADP+ as acceptor

6.2.1.40 - 4-hydroxybutyrate---CoA ligase (AMP-forming)

6.2.1. -acetate---CoA ligase

4.2.1.120 - 4-hydroxybutanoyl-CoA dehydratase

4.2.1.17 - enoyl-CoA hydratase

1.1.1.35 - 3-hydroxyacyl-CoA dehydrogenase

6.4.1.2 - acetyl-CoA carboxylase

1.2.1.75 - malonyl-CoA reductase (malonate semialdehyde-forming)

1.1.1.298 - 3-hydroxypropionate dehydrogenase (NADP+) RoseRS_3201

6.2.1.36 - 3-hydroxypropionyl-CoA synthase

4.2.1.116 - 3-hydroxypropionyl-CoA dehydratase

1.3.1.84 - acrylyl-CoA reductase

Dicarboxylate/4-Hydroxybutyrate cycle

2.7.9.1 - pyruvate, phosphate dikinase

2.7.9.2 - pyruvate, water dikinase

4.1.1.31 - phosphoenolpyruvate carboxylase

1.1.1.37 - malate dehydrogenase

4.2.1.2 - fumarate hydratase

1.3.4.1 - fumarate reductase (CoM/CoB)

1.3.1.6 - fumarate reductase (NADH)

2.8.3.22 - succinyl-CoA---L-malate CoA-transferase

1.2.1.76 - succinate-semialdehyde dehydrogenase (acylating)

1.1.1. -With NAD+ or NADP+ as acceptor

6.2.1.40 - 4-hydroxybutyrate---CoA ligase (AMP-forming)

6.2.1. - acetate---CoA ligase

4.2.1.17 - enoyl-CoA hydratase, crotonyl-CoA hydratase

1.1.1.35 - 3-hydroxyacyl-CoA dehydrogenase

Calvin–Benson–Bassham cycle

4.1.1.39 - ribulose-bisphosphate carboxylase

2.7.1.19 - phosphoribulokinase

2.7.1.14 - sedoheptulose 7-phosphotransferase

4.1.2.13 - fructose-bisphosphate aldolase

3.1.3.37 - sedoheptulose-bisphosphatase

4.1.2.13 - fructose 1,6-diphosphate aldolase

Reversible tricarboxylic acid cycle

2.3.3.1 - citrate synthase
