# Supplementary material for: Diversity and Metabolic Potential of the Terrestrial Mud Volcano Microbial Community with a High Abundance of Archaea Mediating the Anaerobic Oxidation of Methane
Source: Life (Basel). 2021 Sep 11;11(9):953. doi: 10.3390/life11090953 (PMC8470020; doi:10.3390/life11090953)
Supplement: Supplementary file 1 [file life-11-00953-s001.zip › Supplementary Table S6 Cytochromes for proof.pdf]

life

MDPI

Supplementary material of Diversity and Metabolic Potential of the Terrestrial Mud Volcano Microbial Community with a High Abundance of Archaea Mediating the Anaerobic Oxidation of Methane

Table S6. Multiheme c-type cytochromes encoded in MAGs of ANME-3 (KA19) and ANME-2a-2b (KA2).

KA19				KA2			
Locus tag	Number of heme *c*-binding motifs	Length, aa	Predicted cellular location	Locus tag	Number of heme *c*-binding motifs	Length, aa	Predicted cellular location
peg.67	9	206	TM*	peg.2	15	286	TM
peg.68	11	341	TM	peg.24	45	1951	cytoplasm
peg.108	10	311	TM	peg.36	2	380	TM
peg.718	8	190	periplasm	peg.99	26	1206	TM
peg.719	11	279	periplasm	peg.121	16	373	TM
peg.1468	8	288	TM	peg.273	4	151	TM
peg.1469	8	393	TM	peg.322	47	1803	cytoplasm
peg.1470	3	356	TM	peg.502	5	228	periplasm
peg.1643	5	464	cytoplasm	peg.504	4	202	TM
peg.1759	15	379	TM	peg.506	8	295	TM
peg.1953	3	226	TM	peg.558	6	483	TM
peg.1978	4	612	TM	peg.721	9	213	TM
peg.1999	4	261	TM	peg.722	16	367	TM
				peg.952	6	333	TM
				peg.954	4	151	periplasm
				peg.979	8	497	TM
				peg.991	11	304	periplasm
				peg.1169	5	527	TM
				peg.1308	9	311	TM
				peg.1380	5	458	TM
				peg.1471	22	1425	TM
				peg.1581	4	601	TM
				peg.1584	18	1208	TM
				peg.1615	44	2173	TM
				peg.1629	25	1337	TM
				peg.1843	44	2233	TM
				peg.1913	26	1171	TM
				peg.2493	67	6593	TM

*TM – transmembrane
